# ‘Status Quo’ on Different Aspects of Gender Distribution in Rheumatology in Germany—Results from a Nationwide Online Survey among Physicians

**DOI:** 10.3390/jcm12134328

**Published:** 2023-06-27

**Authors:** Sarah Ohrndorf, Martin Krusche, Xenofon Baraliakos, Eugen Feist, Barbara Gundelach, Isabell Haase, Bimba Franziska Hoyer, Uta Kiltz, Michaela Koehm, Anna Julia Voormann, Philipp Sewerin, Johanna Mucke

**Affiliations:** 1 Department of Rheumatology and Clinical Immunology, Charité–Universitätsmedizin Berlin, 10117 Berlin, Germany; 2 Center for Internal Medicine and Rheumatology, Universitätsklinikum Hamburg-Eppendorf, Medical Faculty, Hamburg University, 20251 Hamburg, Germany; 3 Rheumazentrum Ruhrgebiet, Ruhr-University Bochum, 44649 Herne, Germany; 4 Department of Rheumatology and Immunology, HELIOS Clinic Vogelsang-Gommern, 39245 Gommern, Germany; 5 German Society for Rheumatology, 10117 Berlin, Germany; 6 Department of Rheumatology, University Hospital Düsseldorf, Medical Faculty, Heinrich Heine University, 40225 Duesseldorf, Germany; 7 Rheumatology and Clinical Immunology, University Clinic Schleswig-Holstein, Medical Faculty, University Kiel, Campus Kiel, 24105 Kiel, Germany; 8 Department of Translational Rheumatology, Immunology-Inflammation Medicine, University Hospital, Goethe University, 60596 Frankfurt am Main, Germany; 9 Fraunhofer Institute for Translational Medicine and Pharmacology ITMP, 60596 Frankfurt am Main, Germany; 10 Commission for Gender Equity in Rheumatology, German Society for Rheumatology, 10117 Berlin, Germany

**Keywords:** rheumatology, gender equity, gender distribution

## Abstract

**Highlights:**

**What are the main findings?**

- The presented survey was initiated by the Commission for ‘Gender Equity in Rheumatology’ of the German Society for Rheumatology to assess the ‘status quo’ in gender distribution among rheumatologists in Germany. The results of the survey provide insights into different aspects of gender distribution in German rheumatology: **1.** gender distribution at different hierarchical levels, **2.** part time work and career consequences, **3.** care work, and **4.** suggestions for achieving (more) gender balance.
- Regarding the gender ratio at different hierarchical levels, 74% of respondents reported more men than women in leadership positions. Part-time work was possible in the departments of 86% of respondents, with more women working part-time (56%) compared to men (29%). Most respondents stated their impression that employees working part-time did not have the same career chances as full-time workers in their departments. In total, 66% agreed that activities to improve gender equity are necessary. The highest need was seen in reconciling work and family through, e.g., part-time models, flexible childcare options at work and a higher acceptance of part-time work in leadership positions.

**What are the implications of the main findings?**

- According to our results, a gender imbalance is prevalent among rheumatologists in Germany, with lower numbers of women evident at higher hierarchical levels. Traditional role assignments are still represented, for example, by a higher proportion of part-time work in women and by the higher amount of care work carried out by women. To close the gender gap in the future, we propose different possible solutions, e.g., childcare at work with employee-friendly opening hours (24/7) and childcare options for special circumstances, as well as higher acceptance of part-time working men and women in higher hierarchical positions.

**Abstract:**

Objectives: Despite the increasing number of female medical students and fellows in Europe, women are still under-represented in higher academic careers and positions in medicine. The aim of this survey was to assess the ‘status quo’ on gender distribution among rheumatologists in Germany. Methods: A web-based anonymous survey (21 questions with multiple answers and free text) using QuestionPro^®^ was distributed among rheumatologists in Germany via newsletters, social media and personal contact, including questions regarding hierarchical positions and work characteristics. Results: Among the total of 170 respondents (72% women, 28% men, 1% diverse), 48% were rheumatologists in training, 35% were trained rheumatologists and 7% were heads of rheumatology departments. Regarding the gender ratio at different hierarchical levels, 74% of respondents reported more men than women in leadership positions. Part-time work was possible in the departments of 86% of respondents, with more women working part-time (56%) compared to men (29%). Most respondents stated their impression that employees working part-time did not have the same career chances as full-time workers in their departments. In total, 66% agreed that activities to improve gender equity are necessary. The highest need was seen in reconciling work and family through, e.g., part-time models, flexible childcare options at work and a higher acceptance of part-time work in leadership positions. Conclusions: According to our results, a gender imbalance is prevalent among rheumatologists in Germany, with lower numbers of women evident at higher hierarchical levels. Traditional role assignments are still represented by a higher proportion of part-time work in women. The establishment of structural changes to achieve better gender equity is needed.

## 1. Introduction

Despite the increasing number of female medical students and fellows, women are still under-represented in higher career and academic positions in medicine in Europe [1,2].

Rheumatology is considered a gender-equitable and family-friendly specialty of internal medicine not only because of the many women affected by rheumatic and musculoskeletal diseases (RMD), but also due to the relatively low incidence of acute emergency cases and the higher rate of outpatient care, which usually makes it a plannable specialty of internal medicine with higher time flexibility and more part-time opportunities. In the last few years, many women have decided to pursue careers in rheumatology. However, women are still under-represented at higher hierarchical levels, especially in academic and leadership positions in rheumatology [3,4,5,6]. Moreover, amongst the authors of recommendations and scientific guidelines, women are under-represented compared to men [7,8], and less often invited as speakers at annual rheumatology conferences, even in large countries with high academic output, such as Germany or France [9,10].

To improve gender balance in rheumatology in Germany and, in doing so, attract more young fellows and researchers to the field of rheumatology in the future, the Commission for ‘Gender Equity in Rheumatology’ of the German Society for Rheumatology was founded in 2021. Recently, our commission published consented recommendations for “Gender-neutral language in the German Society for Rheumatology (DGRh e.V.)” [11]. The reason for the presented survey was to assess the ‘status quo’ on different aspects of gender distribution among physicians in the field of rheumatology in Germany with the aim of investigating the extent of gender inequity, as well as to discuss, develop and present solutions for the improvement of gender balance to create more diversity between all genders in the future, especially in academic and leadership positions. 

## 2. Methods

A national web-based anonymous survey using QuestionPro^®^ was distributed among rheumatologists in Germany via electronic newsletters (of which 1.624 members were contacted), social media and personal contact between September 2021 and January 2022. The survey was developed by members of the Commission for ‘Gender Equity in Rheumatology’ of the German Society for Rheumatology based on a narrative literature review [4] and consensus among the commission members. It consisted of 21 questions with multiple answers and, in special questions, free text. In total, four different domains were assessed: 1. gender distribution at different hierarchical levels, 2. part time work and career consequences, 3. care work, and 4. suggestions for achieving (more) gender balance. Descriptive statistics (number of responses in %) were performed using R Version 4.2.1.

## 3. Results

### 3.1. Characteristics of the Respondents of the Survey

Among all 170 respondents who completed the survey, 72% (*n* = 122) were women, 28% (*n* = 47) were men and 1% (*n* = 1) was diverse. In total, 73% (*n* = 124) were employed at a rheumatology clinic, with 79% (*n* = 98) working at an academic institution and 20% (*n* = 25) at a non-academic clinic; 1% (*n* = 1) did not specify their institution. A total of 48% (*n* = 59) were rheumatologists in training, 35% (*n* = 44) were trained rheumatologists and 7% (*n* = 8) were heads of rheumatology departments (10% other). 

### 3.2. Gender Distribution at Different Hierarchical Levels for Respondents’ Institutions

**Figure 1:**
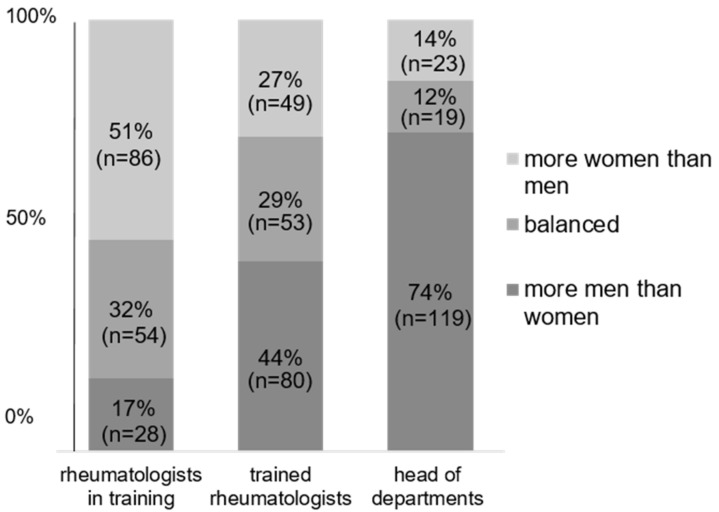
Gender ratio at different hierarchical levels.

Regarding their institutions, 17% of respondents reported more men than women in training at their workplace, and according to 32%, the gender ratio was balanced. At the staff level, more men than women were reported by 44% (according to 29%, the gender ratio was balanced). In leadership positions, 74% reported more men than women heading rheumatology (12% balanced) (Figure 1).

## 4. Part-Time Work and Career Consequences

Part-time work was possible in 86% of respondents’ departments, with women working part-time in 56% of cases, both women and men working part-time in 29%, and only men working part-time in 1%. 

**Figure 2:**
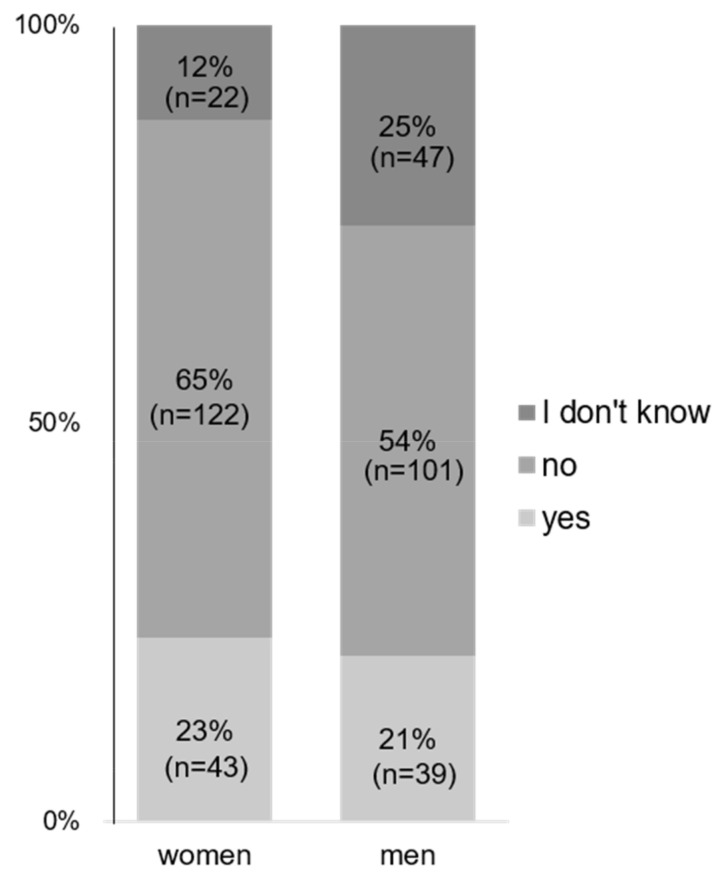
Opportunities for advancement in part-time vs. full-time employees: “Do employees working part time have similar opportunities for advancements as employees working full time?”.

Most respondents stated that according to their perception, men and women working part-time did not have the same opportunities for advancement compared to employees working full-time (Figure 2).

**Figure 3:**
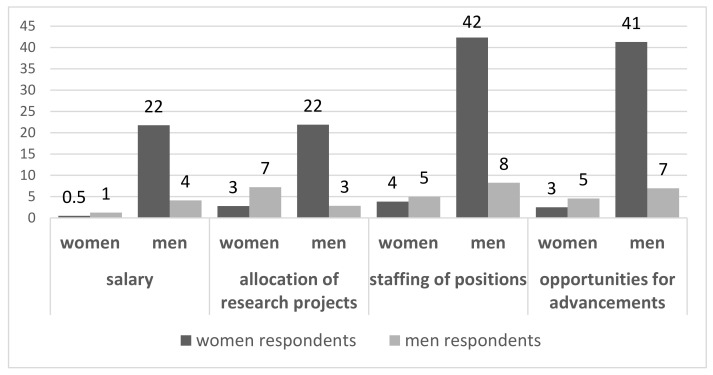
Perceived prioritization of men and women empolyees according to men and women respondents in percent. “In your rheumatology workplace, do you experience that men or women are preferred over the other gender despite equal performance in terms of ...”.

The majority of respondents perceived a preference for a specific gender despite equal performance regarding different work aspects: men were more often perceived as preferred in terms of opportunities for advancement (by 48% of all respondents), the allocation of certain positions in the hierarchy (by 50%), the allocation of research projects (by 25%), and salary (by 26%), as reported by the responding women. (Figure 3). If this was stratified by the gender of those in leadership positions (i.e., more men than women in the top position vs. a balanced gender ratio in the top position vs. more women than men in the top position), men were still seen as more privileged by all respondents, albeit to a lower extent by respondents at institutions with more women than men in higher positions (Supplementary Figure S1).

## 5. Care Work

**Figure 4:**
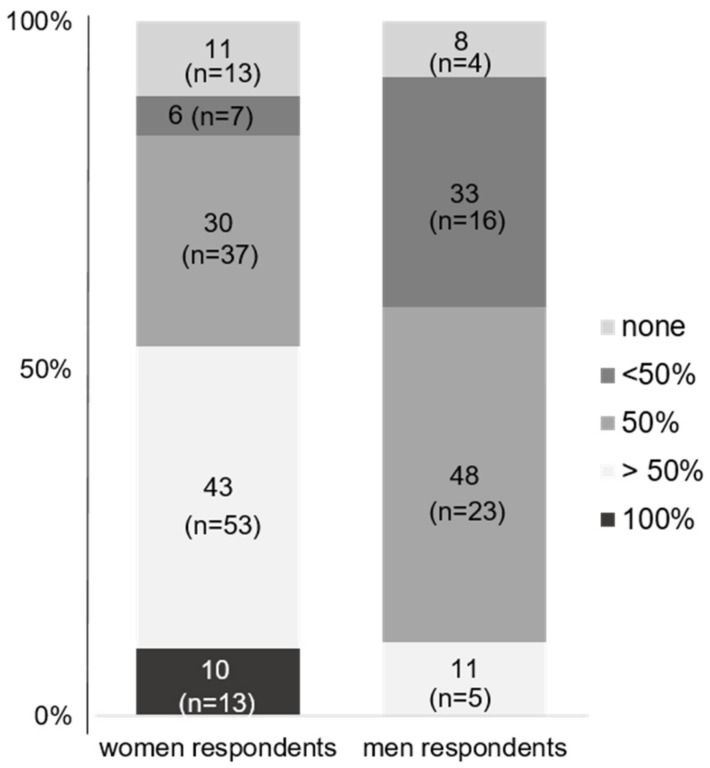
Share of care work by gender in % of total care and family work: “What share of the total care and family work do you perform?”

While 43% of the responding women were responsible for >50% of family care and housework, compared to 11% of the responding men, the majority of responding men stated that they covered 50% (49% of men) or less than 50% (32% of responding men) of family work (Figure 4).

## 6. Suggestions for Achieving Gender Balance

For 66% of respondents, the need for activities to improve gender equity was evident. The highest need was seen for the improvement of the compatibility of care and work through adequate part-time models, childcare options at work, and a higher acceptance of part-time working men and women in higher positions. 

## 7. Discussion

The presented survey was initiated by the Commission for ‘Gender Equity in Rheumatology’ of the German Society for Rheumatology to assess the ‘status quo’ in gender distribution among rheumatologists in Germany and investigate the extent of gender inequity. The results of the survey provide insights into different aspects of gender distribution in German rheumatology, for example, gender differences on the hierarchical ladder. The survey responses, which were predominantly provided by women (72%), show that higher hierarchical positions are predominantly held by men. This finding is in line with similar results from previous studies on gender distribution in rheumatology [3,4,5,6]. Ovseiko et al. recently performed a cross-sectional EULAR survey on gender equity in academic rheumatology, which revealed disproportionately fewer women in academic rheumatology than in clinical rheumatology, and that women tended to be under-represented in senior academic roles [12]. There are plenty of reasons for this misbalance. On one hand, women have more difficulties in being taken seriously than men [13], so that they are assigned tasks that do not help them to advance, and they often experience sexism during their academic careers [14]. Furthermore, there is a positive link between stereotype threat and perceived negative career consequences of utilizing family-friendly policies [15]. 

On the other hand, the majority of women are still responsible for the main part of family care work, making it very hard to work full-time in parallel with their care roles and focus on their career goals, which was also reflected in this survey. Accordingly, the respondents stated that although working part-time was possible in most cases, employees working part-time did not have the same career opportunities as full-time employees (Figure 2). In general, working part-time often leads to exclusion from team gatherings and meetings, which are often held later in the evening and where most of the interaction takes place and important decisions are made. This automatically reduces their career chances compared to full-time employees. 

However, many investigations show that companies with a high number of part-time employees have a similar or even better output [16], a fact that has not yet been recognized in the healthcare sector, where part-time work still means reduced career opportunities. 

Another important aspect is the traditional family model that still exists in Germany, with women often being responsible for care work and men being the breadwinners (Figure 4). To achieve gender equity, men and women must be treated equally in terms both professional and family responsibilities. If men are discriminated against at work when they decide to reduce their working hours to take care of their children, while this is well accepted for women (although still associated with lower career opportunities), gender equity cannot be achieved.

Respondents were also asked to provide suggestions for a more gender-balanced rheumatology society, which includes improvement of the compatibility of care and work through adequate part-time models, childcare options at work, and higher acceptance of part-time working men and women by employers and persons in higher hierarchical positions. 

Therefore, to achieve gender equity, a change of mindset is needed, which can be implemented in several ways: Childcare at work with employee-friendly opening hours (24/7), childcare options beyond regular hours or for special circumstances (weekend shifts, sick children) and (financial) support for childcare at conferences and meetings. Furthermore, employers need to enable flexible working with the option for working part-time. As an example, clinical reports could be written at home, and meetings could either be held before childcare closing hours, online or in a hybrid format. 

The implementation of adequate and non-discriminating part-time models, such as job sharing, should be feasible in rheumatology as it is an outpatient-dominated and plannable specialty. However, to improve career options, time for research during working hours is important and should be supported either by the head of a department or through institutional programs, e.g., clinician scientists. As presented in Supplementary Figure S1, putting more women in leadership positions also has the effect of more gender-balanced advancements, which is in line with the findings by Bosello et al. [6].

The main limitations of the presented results are that more women than men answered the survey, which is an important bias; a more representative analysis should be better gender-balanced. Moreover, the results are based on the perceptions of the respondents, so the survey represents subjective and maybe momentary insights from the respondents. In addition, to ensure data protection, we did not assess the respondents’ workplaces. Thus, there might be a location bias with many answers from single centers. Furthermore, due to the nature of the survey, only descriptive data analysis can be presented. However, the relatively high number of respondents (170 respondents) suggests a broad distribution among several rheumatology departments. 

To summarize, we aim to create awareness of gender inequality in the field of rheumatology in Germany. At the same time, we propose different solutions to close the gender gap in the future through the development and promotion of part-time models and the integration of part-time employees in the clinical and administrative routine. 

However, the achievement of gender equity requires a further change in the societal mindset regarding traditional family models and the optimization of (child)care options. 

## Data Availability

The data that support the findings of this study are available on request from the corresponding author.

## Supplementary Materials

The following supporting information can be downloaded at: https://www.mdpi.com/article/10.3390/jcm12134328/s1, Figure S1: Perceived prioritization of men and women employees stratified by the proportion of women and men in top positions.
