# Supplementary material for: ‘Status Quo’ on Different Aspects of Gender Distribution in Rheumatology in Germany—Results from a Nationwide Online Survey among Physicians
Source: J Clin Med. 2023 Jun 27;12(13):4328. doi: 10.3390/jcm12134328 (PMC10342364; doi:10.3390/jcm12134328)
Supplement: Supplementary file 1 [file jcm-12-04328-s001.zip › jcm-2277716-supplementary.pdf]

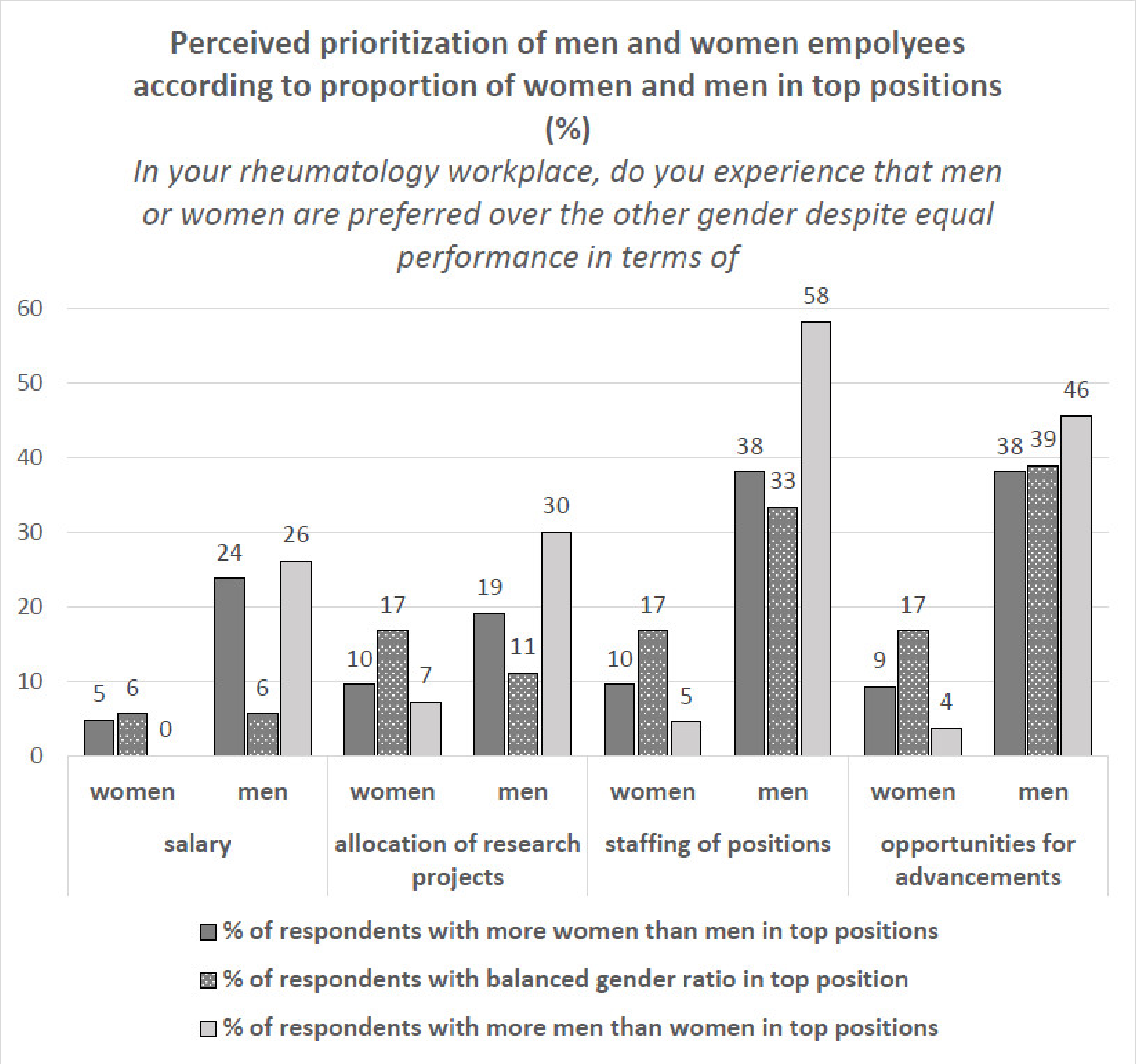


**Figure S1**. Perceived prioritization of men and women employees stratified by the proportion of women and men in top positions.

**Question 1**

What is the numerical ratio of women to men in your rheumatology workplace?

(Please answer only those levels for which you are able to give an answer).

At the level of physicians in further training and comparable positions (in clinics or branches)

- more men than women

- balanced

- more women than men

At the level of specialists, senior physicians, physicians in private practice or comparable positions (in hospitals or private practice)

- more men than women

- balanced

- more women than men

At the level of chief physician, head of medical department or comparable positions (hospital only)

- more men than women

- balanced

- more women than men

**Question 2**

Are there any employees in your direct medical work environment who work part-time?

- No

- Yes, only women

- Yes, more women than men

- Yes, equal number of women and men

- Yes, more men than women

- Yes, only men

- I don't know/can't judge

**Question 3**

In your rheumatology workplace, do you experience that men are given preference over women for equal performance with regard to...?

**Advancement opportunities**

- Men are not preferred at all

- Men are preferred rather little

- Men tend to be preferred more

- Men are strongly preferred

- I don't know/can't judge

**Staffing of positions**

- Men are not preferred at all

- Men are preferred rather little

- Men are preferred more

- Men are strongly preferred

- I don't know/can't judge

**Salary**

- Men are not preferred at all

- Men are preferred rather little

- Men are preferred more

- Men are strongly preferred

- I don't know/can't judge

**Allocation of research projects**

- Men are not preferred at all

- Men are preferred rather little

- Men are preferred more

- Men are strongly preferred

- I don't know/can't judge

**Question 4**

In your rheumatology workplace, do you experience that women are given preference over men for equal performance with regard to...?

**Advancement opportunities**

- Women are not preferred at all

- Women are preferred rather little

- Women tend to be preferred more

- Women are strongly preferred

- I don't know/can't judge

**Staffing of positions**

- Women are not preferred at all

- Women tend to be preferred less

- Women tend to be preferred more

- Men are strongly preferred

- I don't know/can't judge

**Salary**

- Women are not preferred at all

- Women are rather less preferred

- Women are preferred more

- Women are strongly preferred

- I don't know/can't judge

**Allocation of research projects**

- Women are not preferred at all

- Women are preferred less

- Women are preferred more

- Women are strongly preferred

- I don't know/can't judge

**Question 5**

In your opinion, do full-time and part-time employees have the same opportunities for advancement in your workplace?

- Yes

- no

- I don't know/can't say

Women who work part-time have the same opportunities for advancement as women who work full-time.

- Yes

- Yes No

- I don't know/can't say

Men who work part-time have the same opportunities for advancement as men who work full-time

- Yes

- No I don't know/can't say

- I don't know/can't say

**Question 6**

Are meetings and internal training sessions at your workplace organized in such a way that part-time employees can also attend during working hours?

- Yes, always

- Yes, most of the time

- Yes, sometimes

- Never

**Question 7**

In your opinion, are activities needed to ensure equal opportunities for women and men in your work environment?

- Yes, definitely

- Rather yes

- Rather no

- No, definitely not

- I don't know/can't judge

**Question 8**

If you answered "rather no" or "no" to the previous question: Why not?

(answer as free text)

**Question 9**

Which of the following measures exist at your workplace explicitly on the topic of equal treatment and equal opportunities?

Flexibility measures (e.g. part-time models, home office, extra parental leave)

Support for the care of family members

Support for career planning (by superiors, etc.)

Mentoring

Networks

Coaching

Job sharing

Seminars on role distribution/expectations to raise awareness of gender stereotypes

Promotion of gender-neutral language

Gender-neutral evaluation and hiring processes

Emphasis on female role models

Target agreements for quotas for women

Explicit statements by management on equal opportunities

Equal Opportunity Officer

- Measure exists and is actively offered

- Measure exists, but is only introduced/implemented on request

- Measure exists in theory

- Measure does not exist

- I don't know/can't judge

**Question 10**

In your opinion, what activities would be important when it comes to ensuring gender equality in your work environment? (Answer as free text)

**Question 11**

Do you have any other comments about gender equality in your workplace that you would like to share? (Answer as free text)

**Part II: Question 12**

In the German Society for Rheumatology (DGRh)In your opinion, is there equal opportunity between women and men when filling positions in the committees of the DGRh?

- Yes, definitely

- Rather yes

- Rather no

- No, definitely not

- I don't know/can't judge

**Question 13**

In your opinion, should the DGRh ensure balance when filling positions on its committees, for example by introducing a quota for women?

- Yes

- No

- I don't know/can't judge

**Question 14**

If yes, how high do you think such a quota for women should be?

- 50%

- 30%

- other

**Question 15**

If you answered "other": Which women's quota do you propose? (Answer as free text)

**Question 16**

Do you think an equal opportunity commission in rheumatology would be useful?

- Yes, definitely

- No, it is not necessary

- I am undecided

- I cannot judge

**Question 17**

What would you like to see from the Equal Opportunities Commission of the DGRh? (Answer as free text)

**Question 18**

In your opinion, should the DGRh develop an equal opportunities concept to ensure equal opportunities in the professional society?

- Yes, this is a good idea

- No, not necessary

- Don't know / no answer

**Question 19**

Do you have any other comments on gender equality in the DGRh that you would like to share? (Answer as free text)

**Question 20**

I am

- Female

- Male

- diverse

**Question 21**

I am a DGRh member

- Yes

- No
